# Six-minute walking distance in healthy Chinese people older than 60 years

**DOI:** 10.1186/s12890-020-01211-w

**Published:** 2020-06-22

**Authors:** He Zou, Jia Zhang, Yingying Zou, Xiaoshu Chen, Yi Wang, Hao Chen, Fanhao Ye, Haizhu Yu

**Affiliations:** 1 Department of Cardiovascular Medicine, Wenzhou People’s Hospital, the Wenzhou Third Clinical Institute Affiliated with Wenzhou Medical University, Wenzhou, Zhe Jiang China; 2 Department of Inspection Medical, Wenzhou People’s Hospital, the Wenzhou Third Clinical Institute Affiliated with Wenzhou Medical University, Wenzhou, Zhe Jiang China; 3 Digestive System Department, The Third Affiliated Hospital of Qiqihar Medical College, Qiqihar, Heilongjiang China; 4 Department of General Practice, Zhejiang Hospital, Hangzhou, Zhe Jiang China

**Keywords:** Six-minute walking test, Reference equation, Exercise testing, Healthy people

## Abstract

**Background:**

The six-minute walking test (6MWT) is a tool that plays a key role in evaluating the functional exercise capacity, prognosis and evaluation of treatment response of patients with various cardiopulmonary diseases. However, standard reference equations are currently unavailable for the six-minute walking distance (6MWD) for people aged 60–85 years in China. The purpose of this study was to 1) measure the 6MWD of healthy Chinese people aged 60–85 years, 2) establish reference equations for predicting the 6MWD, and 3) compare our reference equations with equations reported in previously published studies.

**Method:**

We obtained informed consent from each participant prior to the test, and the research design was approved by the Ethics Committee of Wenzhou People’s Hospital. The demographic and anthropometric data and the 6MWD of healthy Chinese subjects aged 60–85 years old were measured using a standardized protocol. Every subject completed two 6MWTs, and the longest 6MWD further analyzed.

**Results:**

Two hundred sixty-six subjects (128 males and 138 females) completed the 6MWT, and the mean walking distance was 518 ± 72 m. Males achieved a longer walking distance than females (518 ± 72 m vs. 487 ± 70 m; *p* < 0.0001), and active subjects achieved a longer walking distance than nonactive subjects (512 ± 76 m vs. 485 ± 63 m; *p* < 0.0001). According to the univariate analysis, the 6MWD was significantly associated with age, height, body mass index (BMI), heart rate and blood pressure after exercise and changes in heart rate before and after exercise. The stepwise multivariate regression analysis identified age, height and BMI as independent predictors of the 6MWD. The reference equations for Caucasians and South Americans tended to overestimate the 6MWD of our subjects, while the equations for Asian and African populations tended to underestimate the 6MWD.

**Conclusions:**

This study is the first to describe the 6MWD of healthy Chinese people aged 60–85 years, and reference prediction equations were proposed. These findings will help to improve the evaluation of Chinese patients with diseases that affect exercise capacity.

## Background

Walking capacity is an effective, sensitive, and reliable measure that is suitable for evaluating and monitoring the functional status and overall health of a wide range of people. These capabilities have led to the designation of walking speed as the “sixth vital sign” [1]. Similar to other vital signs, walking speed is a simple assessment that provides valuable information about potential physiological processes. The six-minute walking test (6MWT) is an exercise test protocol that is recommended for use in a serial manner to assess changes in exercise capacity over time, in response to medical treatment and/or training [2]. Since the publication of the previous American Thoracic Society (ATS) statement on the 6MWT in 2002 [3], new information regarding the 6MWT has appeared in many areas, including methods for assessing test performance and interpretation. In research and clinical settings, the improvement of this knowledge system has significant implications for the good conduct of the 6MWT in individuals with chronic cardiopulmonary diseases. When we apply reference equations for 6MWD to individuals, a significant change in the predicted values will occur. If reference values are applied, the equations generated and verified in the local population should be used when possible. Therefore, reference equations for the 6MWD of different populations have been produced by numerous researchers [4–12]. In previous studies, we established reference equations for the 6MWD of healthy Chinese individuals aged from 18 to 59 years [13]. However, many patients are usually affected by cardiopulmonary disease when they are more than 60 years old. These special populations must accept the 6MWT, which has been confirmed to improve quality of life, physical function and survival [14]. The lack of a reference value for the 6MWD based on healthy subjects limits the interpretation of the 6MWD in patients and poses problems for clinicians who wish to evaluate patients without disease using the 6MWD.

The purpose of this study was to 1) measure the 6MWD of healthy Chinese people aged 60–85 years, 2) establish reference equations for predicting the 6MWD, and 3) compare our reference equations with the equations obtained in previously published studies.

## Methods

### Subjects

We recruited subjects aged 60 to 85 years to participate in this cross-sectional study and collected data over a 59-month period from December 2013 to October 2018. Prior to the test, we obtained informed consent from all subjects, and the design of the study was approved by the Ethics Committee of Wenzhou People’s Hospital. The subjects included individuals from local universities for the elderly, healthy people in the local community and individuals undergoing physical examinations at public hospitals. Before recruiting the subjects, the researchers informed them of the purpose of the study and asked them to complete a questionnaire to ensure that the subjects were healthy. The researchers also verified the answers to the questionnaire before the subjects conducted the 6MWT.

The following inclusion criteria were established: healthy subjects, subjects with an 18 kg/m^2^ ≤ BMI < 30 kg/m^2^ and subjects aged 60–85 years. The exclusion criteria included subjects with preexisting limitations of exercise ability (muscle, joint or nerve involvement), any organic disease, basal systolic blood pressure ≥ 140 mmHg or diastolic blood pressure ≥ 90 mmHg, basal heart rate < 50 bpm or ≥ 100 bpm, abnormal lung function (FEV_1_ < 80% predicted or FEV_1_/FVC < 70%), extreme weight (BMI < 18 kg/m^2^ or BMI > 30 kg/m^2^) and respiratory symptoms during the past 4 weeks.

### Procedure

Subjects were asked to avoid alcohol, caffeine and large meals for at least two hours before the test, as well as strenuous exercise for 24 h before the test. The age, height, weight and BMI of the subjects were measured before the test. Age was verified using their identity card. When the subject’s back was straight, an altimeter was used to measure the height without shoes. The body weight (kg) was measured with an electronic scale, and the BMI of each subject was calculated as BMI = weight/height^2^ (expressed in kg/m^2^). The smoking history and blood pressure were recorded. According to the recommendations of the ATS [15], lung function was measured using a standard portable lung function meter (Vitalograph Alpha, Ireland).

### Questionnaire assessment

The health state of the participants was assessed using a standardized questionnaire asking the following information: “Are you healthy?”, “Do you have high blood pressure, diabetes or another chronic disease?”, “Are you taking regular medication?”, “Can you participate in physical activities?”, and “Do you have problems with walking or do you need walking aids while walking?”. In addition, we also asked the subjects if they had participated in physical activities with the following question: “How many hours do you participate in exercise activity in a week?”. Subjects were classified as “physically active subjects” if they had performed lower extremity exercises for at least 20 min 3 times per week in the last month [16]. In contrast, if the subjects did not meet this criterion, they were classified as “sedentary subjects”.

### Six-minute walking test

The 6MWT is a self-paced test of walking capacity. The 6MWT was conducted strictly in accordance with the standardized process proposed by the ATS [3]. The 6MWT was completed in a flat, straight 30-m course with a hard surface and little pedestrian traffic. The operator made a mark on the corridor every 3 m. An orange traffic cone was placed at the corner of the 30-m corridor as a marker. The starting line that represented the beginning and end of every 60 m was marked with brightly colored tape. The 6MWT was completed between 9:00 a.m. and 16:00 p.m. and the temperature ranged from 20 to 25 °C to minimize the effects of biological rhythms and temperature. The subjects were asked to sit in a chair near the starting line for at least 10 min prior to the test. Resting blood pressure, heart rate, and oxygen saturation were measured and recorded before the test. We provided the subjects with standardization instructions for the 6MWT, and informed them that aim of this test was to walk as far as possible for 6 minutes without running or jogging. They were allowed to walk along this corridor between the markers, which can be passed many times within 6 minutes. We notified them when each minute was complete, and then allowed them to stop where they were on the course at the end of 6 minutes. Six minutes is a long time to walk, and thus the subjects would be exerting themselves. If the subjects experienced symptoms such as chest pain, leg cramps, dizziness or dyspnea, they were able to stop and rest for a while, but once they recovered, they were encouraged to continue walking as soon as possible. The 6MWT was monitored by a specific operator who recorded the time and measured distance using an electronic timer and a 30-m scale. The subjects were encouraged every minute using standard phrases (i.e., 1 min: “You are doing well, you have five minutes to go”; 2 min: “Keep up the good work, you have four minutes to go”; 3 min: “You are doing well, you are halfway”; 4 min: “Keep up the good work, you have only two minutes left”; 5 min: “You are doing well, you have only one minute to go”; and 6 min: “Please stop where you are”). The 6MWD is the primary result of the 6MWT. The number of laps and the number of meters in the final partial lap were recorded. The total distance walked was calculated and rounded to the nearest meter. If the subjects stopped during the test, the total time they stopped and the number of stops over the 6 minutes were also recorded [17]. Blood pressure, heart rate and oxygen saturation of the subjects were measured and recorded immediately after the test. The subjects were also asked to complete the Borg dyspnea scale to assess their extent of dyspnea after the test was completed [18]. The Borg dyspnea scale is ranked from 0 for no dyspnea and 10 for maximum dyspnea. Each subject completed two 6MWTs that were monitored by a specific assessor. The two 6MWTs were performed at intervals of 2 hours, and the longer 6MWD was used for further analysis.

### Quality assurance

The consistency of test procedures and conditions is essential to ensure the quality of 6MWT results. All assessors must be familiar with the test procedures, as the test must follow a clear process.

## Data analysis

The Kolmogorov-Smirnov test was used to assess the normality of the data. Data are presented as means ± standard deviations (SD) or numbers and percentages, as appropriate. The characteristics of the subjects were analyzed using a descriptive analysis. Paired sample Student’s *t*-tests were used to compare activity and sex between the two groups, and unpaired samples Student’s *t*-test were used to compare the two 6MWDs. The comparisons between the measured 6MWD in our subjects and the 6MWD based on previously published reference equations derived from studies conducted in other countries [5, 10, 11, 19–21] were performed using paired *t*-tests. The repeatability of the two 6MWTs was examined by calculating the intraclass correlation coefficient (ICC) and performing the Bland Altman analysis [22]. We first performed a univariate analysis with Spearman’s correlation test to evaluate the correlations between the 6MWD and the variables, and then conducted a forward stepwise multivariate regression analysis to establish the reference equations for the 6MWD. The most significant categorical variable was added to the model at each step, and the process continued until no additional statistically significant variables were added. A *p-*value> 0.05 was used to determine whether a variable was entered and removed. Data were analyzed using SPSS for Windows statistical software (version 17.0; SPSS, Inc., Chicago, IL). A *p-*value< 0.05 was considered significant in all analyses.

## Results

### Demographic characteristics and 6MWT results

Three hundred thirty-two healthy subjects were recruited for this study. Sixty-six subjects were excluded from the study (foot sprain: n = 1; heart disease: n = 10; basal heart rate < 50 bpm or basal heart rate ≥ 100 bpm: n = 10; hypertension: n = 23; BMI < 18 kg/m^2^ or ≥ 35 kg/m^2^: n = 10; abnormal lung function: n = 6 and diabetes mellitus: n = 6). Finally, 266 subjects (138 women and 128 men) completed the 6MWTs, with no subjects prematurely terminating the test or requiring rest during the test. The characteristics and 6MWT results of all subjects are summarized in Table 1. Males were significantly taller and heavier than females, and differences in BMI were observed between the sexes. The mean 6MWD for all subjects was 502 ± 73 m. The mean distance was 518 ± 72 m for males and 487 ± 70 m for females, and the difference was significant (*p* < 0.001). More than half of healthy subjects had performed lower extremity exercise for at least 20 min, 3 times per week in the last month. The mean distance walked was 512 ± 76 m for physically active subjects and 485 ± 63 m for sedentary subjects, and the physically active subjects walked significantly longer distances than sedentary subjects (*p* < 0.05). The subjects reached approximately 69% of their maximum predicted heart rates (mHRs) at the end of the test. We observed a significant difference in oxygen saturation, resting and post exercise heart rate, and mHRs at the end of the test between the sexes. The mean walking distances of the first and second test sessions were 482 ± 71 m and 489 ± 76 m, respectively.

**Table 1:** Demographic characteristics and 6MWT results of all subjects

Characteristic	Male (n = 128)	Female (n = 138)	*p-*value*	Total (n = 266)
Age, years	72 ± 8	72 ± 8	NS	72 ± 8
Height, cm	167 ± 5	156 ± 5	< 0.001	162 ± 8
Weight, kg	67 ± 7	56 ± 7	< 0.001	61 ± 9
BMI, kg/m^2^	24 ± 2	23 ± 2	< 0.05	24 ± 2
HRi, bpm	75 ± 9	77 ± 10	< 0.05	76 ± 10
HRf, bpm	100 ± 12	104 ± 12	< 0.05	102 ± 12
HR change, bpm	25 ± 12	27 ± 11	NS	26 ± 12
% mHR	68 ± 8	71 ± 8	< 0.05	69 ± 8
SBPi, mmHg	131 ± 8	131 ± 7	NS	131 ± 7
DBPi, mmHg	76 ± 8	78 ± 8	NS	77 ± 8
SBPf, mmHg	155 ± 12	155 ± 12	NS	155 ± 12
DBPf, mmHg	86 ± 9	85 ± 8	NS	86 ± 9
SpO_2_i, %	98 ± 1	98 ± 1	< 0.05	98 ± 1
SpO_2_f, %	97 ± 1.	98 ± 1	< 0.05	97 ± 1
Borg	3 ± 1	3 ± 1	NS	3 ± 1
6MWD, m	518 ± 72	487 ± 70	0.001	502 ± 73

Values are presented as means±SD

*p-values for the comparison between males and females

6MWT (D): six-minute walking test (distance); BMI: body mass index; i: initial; f: final; change: change with exercise; SBP: systolic blood pressure; DBP: diastolic blood pressure; HR: heart rate; SpO_2_: oxygen saturation; % mHR: percentage of the predicted maximum heart rate; SpO_2_i: resting SpO_2_; SpO_2_f: end-test SpO_2_; Borg: dyspnea perception scale

### Associations with the 6MWD

The relationships between the 6MWD and the variables in the males and females are summarized in Table 2. According to the univariate linear regression analysis, the variables (i.e., age, height, BMI, heart rate and blood pressure after the test and change in heart rate with exercise) showed a significant correlation with the 6MWD. The variables age, height and BMI were included in the stepwise multivariate regression analysis. Age, height and BMI were identified as independent factors that influenced the 6MWD and explained approximately 33% of the variance in distance (Table 3). The reference equations for the 6MWD in the males and females are as follows:

**Table 2:** Univariate correlation coefficients for the 6MWD and subject variables

Variable	Male (n = 128)		Female (n = 138)	
	r-value	*p-*value	r-value	*p-*value
Age, years	−0.45	< 0.001	−0.47	< 0.001
Height, cm	0.36	< 0.001	0.36	< 0.001
Weight, kg	−0.004	NS	−0.03	NS
BMI, kg/m^2^	−0.25	< 0.05	− 0.29	< 0.001
HRi, bpm	−0.08	NS	−0.05	NS
HRf, bpm	0.26	< 0.05	0.27	< 0.05
HR change, bpm	0.31	< 0.001	0.34	< 0.001
SBPi, mmHg	−0.04	NS	−0.04	NS
DBPi, mmHg	0.05	NS	0.03	NS
SBPf, mmHg	0.18	< 0.05	0.20	< 0.05
DBPf, mmHg	0.16	< 0.05	0.19	< 0.05
SpO_2_i, %	0.02	NS	0.05	NS
SpO_2_f, %	0.01	NS	−0.02	NS

*6MWD* Six-minute walking distance; r-value: Pearson’s correlation coefficient; *BMI* Body mass index;

*i* Initial, *f* Final, *change* Change with exercise, *SBP* Systolic blood pressure, *DBP* Diastolic blood pressure, *HR* Heart rate, *SpO*_*2*_ Oxygen saturation, *SpO*_*2*_*i* Resting SpO_2_, *SpO*_*2*_*f* End-test SpO_2_;

**Table 3:** Stepwise multivariate linear regression analysis of factors associated with the 6MWD stratified by sex

	Male			Female		
	Unstandardized Coefficient	SE	*p-*value	Unstandardized Coefficient	SE	*p-*value
Constant	233.205	190.385	NS	285.011	184.377	NS
Age, year	−4.010	0.703	< 0.001	−3.506	0.678	< 0.001
Height, cm	4.310	0.998	< 0.001	3.968	1.041	< 0.001
BMI, kg/m^2^	−6.140	2.402	< 0.05	−7.179	2.189	< 0.05
R square	0.346			0.343		
Change in R square	0.33			0.328		

$$ {\displaystyle \begin{array}{l}\mathrm{Male}:6\mathrm{MWD}\left(\mathrm{m}\right)=233.205+\left(4.31\times \mathrm{height}\right)-\left(4.01\times \mathrm{age}+6.14\times \mathrm{BMI}\right);{\mathrm{r}}^2=0.33.\\ {}\mathrm{Female}:6\mathrm{MWD}\left(\mathrm{m}\right)=285.011+\left(1.041\times \mathrm{height}\right)-\left(0.678\times \mathrm{age}+2.189\times \mathrm{BMI}\right);{\mathrm{r}}^2=0.328.\end{array}} $$

### Comparison with published regression equations

Comparisons between the measured 6MWD in our subjects and predicted 6MWD for the same age ranges using previously established reference equations for South American [9, 19], African [21], Caucasian [4, 5] and Asian [20] populations are shown in Table 4. The 6MWD in our subjects was overestimated by four of the reference equations derived from Caucasian and South American populations, including the equations established by Jenkins et al. [4], which overestimated the distance walked by our subjects by 67 ± 60 m (*p* < 0.001), Iwama et al. [11], which overestimated the distance by 16 ± 68 m (*p* < 0.001), Osses et al. [19], which overestimated the distance by 53 ± 62 m (*p* < 0.001) and Troosters et al. [5], which overestimated the distance by 30 ± 64 m (*p* < 0.001). However, the 6MWD of our subjects was underestimated by two reference equations derived from Asian and African populations by Poh et al. [20], which overestimated the distance by 12 ± 64 m (*p* < 0.001) and Fernandes et al. [21], which overestimated the distance by 93 ± 61 m (*p* < 0.001).

**Table 4:** Measured and predicted 6MWD for the same age range from the equations derived in previous studies

Equation	Measured 6MWD (m)	Predicted 6MWD (m)	Predicted-Measured(m)
Jenkins et al.	502 ± 73	569 ± 44	67 ± 60*
Iwama et al.	504 ± 73	520 ± 33	16 ± 68*
Poh et al.	502 ± 73	490 ± 60	−12 ± 64*
Fernandes et al.	526 ± 64	433 ± 24	−93 ± 61*
Osses et al.	511 ± 72	563 ± 39	53 ± 62*
Troosters et al.	511 ± 72	540 ± 62	30 ± 64*

*p < 0.05 using Student’s t-test

*6MWD* Six-minute walking distance

## Discussion

**Fig. 1:**
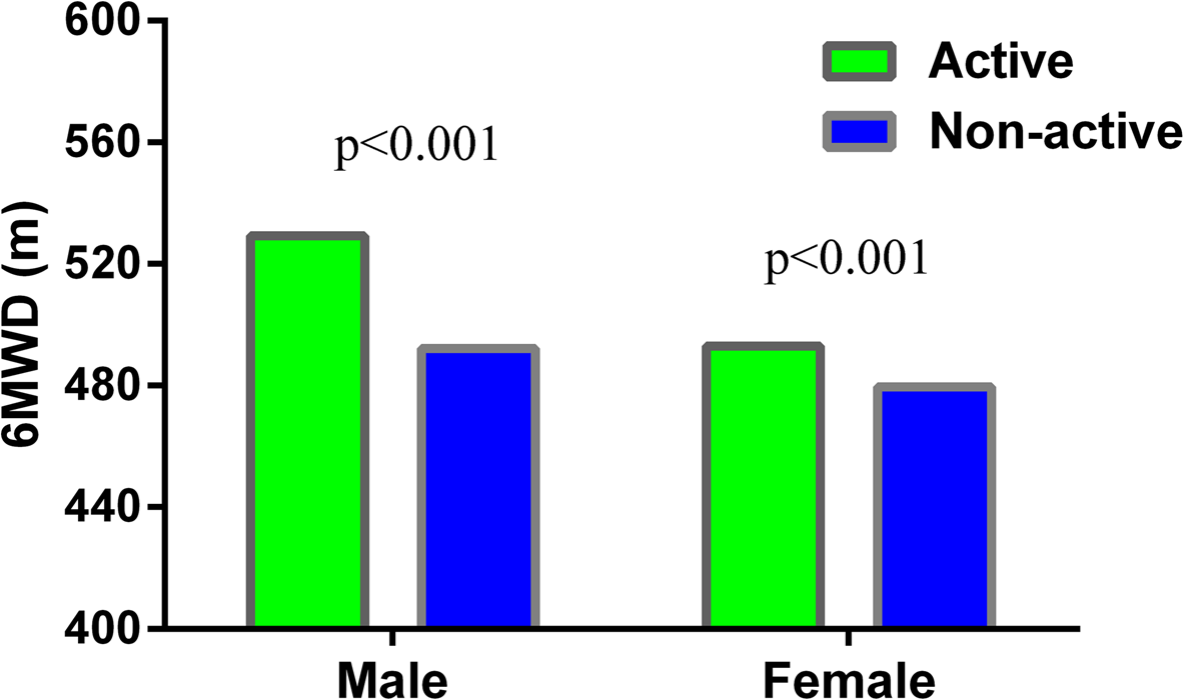
Relationship between the 6MWD and activity in the females and males

To our knowledge, this study is the first to predict the 6MWD in healthy Chinese people aged 60–85 years. A significant difference in the 6MWD was observed between the males and females. Males exhibited a longer 6MWD than females, possibly because males are taller and have a higher level of physical activity and greater muscle mass than the females. In our study, a significant difference in the 6MWD was observed between physically active subjects and sedentary subjects. Figure 1 shows the effect of the activity level on the 6MWD between the males and females. Exercise physiology studies have identified a significant positive correlation between physical exercise and muscle strength [23]. In contrast, sedentary lifestyles usually affect muscle metabolism, muscle quality and physical fitness, which might explain why the 6MWD of sedentary subjects was significantly shorter than physically active subjects in our study [23].

Age was negatively correlated with the 6MWD, which was one of the main variables included in the regression equations. This correlation is probably attributed to the gradual decrease in the muscle mass, muscle strength and maximum oxygen uptake of the person with age. Height was positively correlated with the 6MWD and was one of the main variables included in the regression equations. This correlation may be attributed to a positive correlation between height and stride, which makes walking more effective and results in increased distances. We expected weight to be an important predictor of the 6MWD because overweight may affect gait and increase workload. However, our findings did not support this hypothesis. The potential explanation may be that the subjects in our study were healthy people who were not generally underweight or overweight. Additionally, BMI was negatively correlated with the 6MWD, which was one of the main variables included in the regression equations. This finding might be attributed to the effect of height on the 6MWD.

In the present study, the heart rate after the test and the changes in heart rate before and after the test were significantly positively correlated with the 6MWD, potentially because the heart rate recorded after the test and the changes in heart rate before and after the test represent the level of effort expended during the 6MWT. In addition, we observed a significant difference in pulse oxygen saturation between males and females, potentially because some male subjects smoked, which reduced their pulse oxygen saturation.

**Fig. 2:**
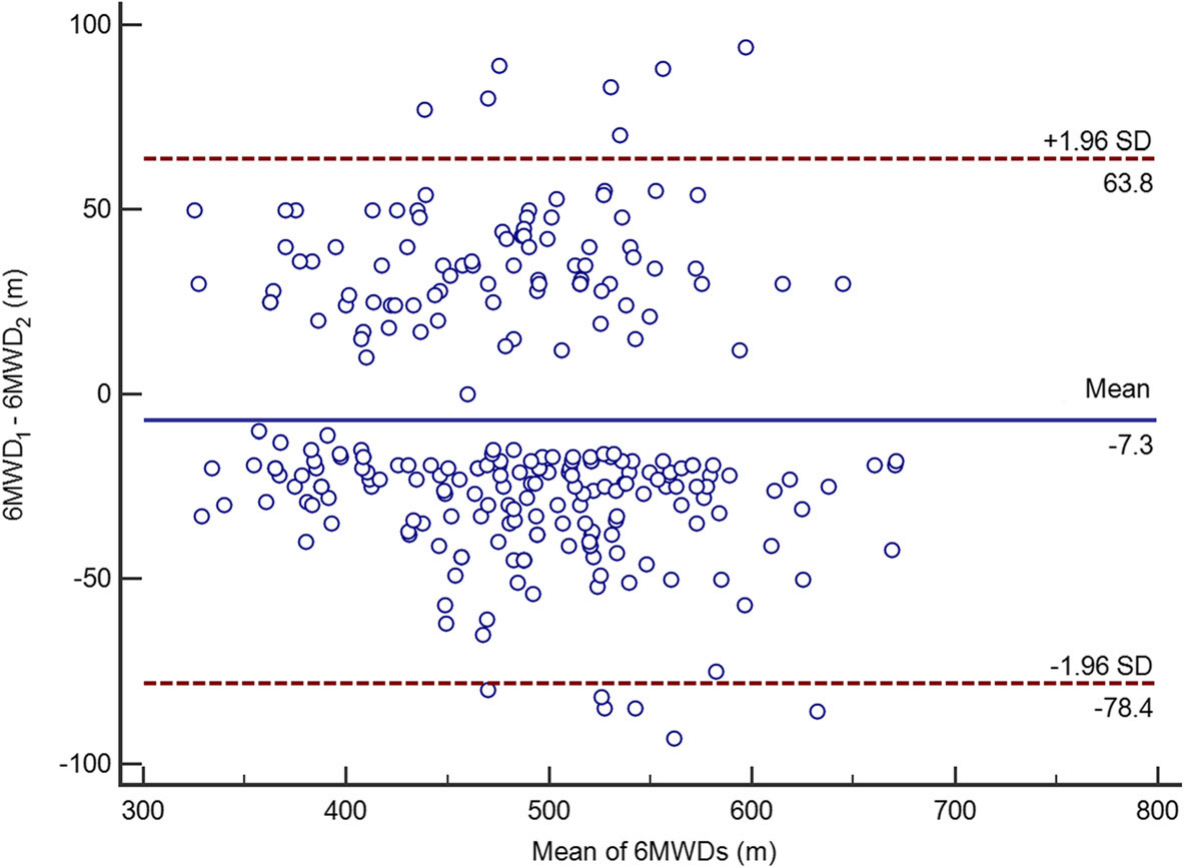
Bland-Altman plot of the results for performance in the first and second 6MWTs

By measuring the 6MWT performance on 2 occasions, the 6MWD during the second test session was longer than the 6MWD during the first test session. The increase in distance may be due to the effect of learning on the 6MWD, such as improving coordination, overcoming anxiety and finding the best gait. Although the walking distance during the second test was increased compared to the first test, the reliability of the 6MWT was good (ICC = 0.87). Previous studies have confirmed the reliability of the 6MWT, consistent with the findings of our study [24]. The Bland-Altman plot showed the mean difference in performance between the first and second 6MWTs (Fig. 2). Thirteen participants had error values outside the 95% confidence interval (CI), and six subjects presented an increase in the second 6MWT, which might have been caused by familiarization with the test. Seven subjects presented a decreased distance in the second 6MWT, which might be due to higher performance on the first 6MWT that led to greater fatigue during the second test.

The comparisons between the measured 6MWD in our subjects and the predicted 6MWD for the same age ranges are based on previous reference equations reported in other studies [4, 5, 10, 11, 20, 21]. These studies were conducted using a variety of populations and methods. The application of these equations to an individual causes substantial variation in the predicted distance. The reference equations for populations in the studies by Troosters et al. [5], Jenkins et al. [4], Iwama et al. [11] and Osses et al. [19] tended to overestimate the 6MWD in our subjects, while the reference equations for populations in studies by Fernandes et al. [21] and Poh et al. [20] populations tended to underestimate the 6MWD. These differences are likely due to differences in the populations and racial backgrounds between samples, as well as the standardized operating procedures used in each study. Our subjects were shorter and had a lower body weight than the subjects studied by Troosters et al. [5], Jenkins et al. [4], Iwama et al. [11] and Osses et al. [19]. Compared with the subjects studied by Poh et al. [20], our subjects were slightly taller and had a greater body weight. Compared with the subjects studied by Fernandes et al. [21], our subjects were slightly taller and had a lower body weight. These differences also emphasize the importance of developing reference equations for the 6MWD for specific populations. The 6MWD is highly sensitive to changes in the method. According to the ATS guidelines, some technical requirements, specifically a 30-m-long corridor and a practice walk, were established to ensure proper execution of the 6MWT. However, some studies did not consider the differences in these technical aspects of the 6MWT. The layout and length of the track will also affect the performance of the test, particularly when using very short track lengths [19]. A longer corridor allows the subjects to spend less time reversing direction, resulting in a longer 6MWD, while a shorter corridor produces exactly the opposite result. In our study, two 6MWTs were performed and the best 6MWD was used for further analysis. The effect of learning on the 6MWD might improve coordination, establish the optimal stride length, and reduce anxiety, thus resulting in a longer 6MWD [25]. In practice, when the 6MWD is used to assess the response to treatment or changes over time, the effect of learning on 6MWD is sufficiently large to be of clinical importance. In some situations, the 6MWT should be performed twice and the best 6MWD recorded. When the 6MWD is used as a one-off measure to stage the disease or assess the risk (such as the likelihood of hospitalization or mortality), the magnitude of the learning effect may not be as important. However, when the 6MWD is close to the predetermined threshold on which the treatment decision is based, the clinician should pay attention to the learning effect. In this case, the repeated testing should be considered. Our subjects reached an average of 69% of their mHRs, but the subjects in the studies by Osses et al. [19], Jenkins et al. [4], Poh et al. [20], and Troosters et al. [5] reached higher mHRs. In contrast, the subjects in the studies by Iwama et al. [11] and Fernandes et al. [21] reached lower mHRs than our subjects. The mHRs also represents the level of effort expended by the subject while performing the test. In addition, the attitudes and psychological factors of the subjects may also influence the 6MWD [26].

Our study has some limitations. First, although the current study included a relatively large sample size, it was a convenience sample rather than a random sample. Second, we did not recruit overweight people, and our reference equations are not applicable to subjects with a BMI > 30 kg/m^2^. A large multicenter study is needed to address these limitations.

## Conclusions

In the present study, the 6MWD of healthy Chinese people aged 60–85 years old was described for the first time, and the prediction reference equations were proposed. The prediction equations containing age, height and BMI explained approximately 33% of the variance in the 6MWD. These equations will help to improve the evaluation of Chinese patients with diseases that affect exercise capacity.

## Data Availability

The data that support the findings of this study are available from Wenzhou People’s Hospital but restrictions apply to the availability of these data, which were used under license for the current study, and thus are not publicly available. However, data are available from the authors upon reasonable request and with the permission of Wenzhou People’s Hospital.

## Abbreviations

6MWT: Six-minute walk test
6MWD: Six-minute walk distance
BMI: Body mass index
HR: Heart rate
SpO_2_: Oxygen saturation
SBP: Systolic blood pressure
DBP: Diastolic blood pressure
ICC: Intra-class correlation
SD: Standard deviation
SPSS: Statistical Package for the Social Sciences
